# Highly efficient Fe^3+^-doped A_2_*BB*′O_6_ (*A* = Sr^2+^, Ca^2+^; *B*, *B*′ = In^3+^, Sb^5+^, Sn^4+^) broadband near-infrared-emitting phosphors for spectroscopic analysis

**DOI:** 10.1038/s41377-022-00803-x

**Published:** 2022-04-27

**Authors:** Dongjie Liu, Guogang Li, Peipei Dang, Qianqian Zhang, Yi Wei, Lei Qiu, Maxim S. Molokeev, Hongzhou Lian, Mengmeng Shang, Jun Lin

**Affiliations:** 1 State Key Laboratory of Rare Earth Resource Utilization, Changchun Institute of Applied Chemistry, Chinese Academy of Sciences, 130022 Changchun, China; 2 University of Science and Technology of China, 230026 Hefei, China; 3 Faculty of Materials Science and Chemistry, China University of Geosciences, 430074 Wuhan, China; 4 Zhejiang Institute, China University of Geosciences, 311305 Hangzhou, China; 5 Laboratory of Crystal Physics, Kirensky Institute of Physics, Federal Research Center KSC SB RAS, Krasnoyarsk, 660036 Russia; 6 Institute of Engineering Physics and Radioelectronics, Siberian Federal University, Krasnoyarsk, 660041 Russia; 7 Research and Development Department, Kemerovo State University, Kemerovo, 650000 Russia; 8 School of Material Science and Engineering, Shandong University, 266071 Jinan, China

**Keywords:** Inorganic LEDs, Optical properties of diamond

## Abstract

Near-infrared (NIR)-emitting phosphor-converted light-emitting diodes have attracted widespread attention in various applications based on NIR spectroscopy. Except for typical Cr^3+^-activated NIR-emitting phosphors, next-generation Cr^3+^-free NIR-emitting phosphors with high efficiency and tunable optical properties are highly desired to enrich the types of NIR luminescent materials for different application fields. Here, we report the Fe^3+^-activated Sr_2−*y*_Ca_*y*_(InSb)_1−*z*_Sn_2*z*_O_6_ phosphors that exhibit unprecedented long-wavelength NIR emission. The overall emission tuning from 885 to 1005 nm with broadened full-width at half maximum from 108 to 146 nm was realized through a crystallographic site engineering strategy. The NIR emission was significantly enhanced after complete Ca^2+^ incorporation owing to the substitution-induced lower symmetry of the Fe^3+^ sites. The Ca_2_InSbO_6_:Fe^3+^ phosphor peaking at 935 nm showed an ultra-high internal quantum efficiency of 87%. The as-synthesized emission-tunable phosphors demonstrated great potential for NIR spectroscopy detection. This work initiates the development of efficient Fe^3+^-activated broadband NIR-emitting phosphors and opens up a new avenue for designing NIR-emitting phosphor materials.

## Abstract (web summary)

Highly efficient Fe^3+^-activated Sr_2-*y*_Ca_*y*_(InSb)_1-*z*_Sn_2*z*_O_6_ near-infrared-emitting phosphors exhibit tunable emission from 885 to 1005 nm with cation substitution of Ca^2+^ for Sr^2+^ and further cosubstitution of [Sn^4+^–Sn^4+^] for [In^3+^–Sb^5+^].

## Graphical abstract

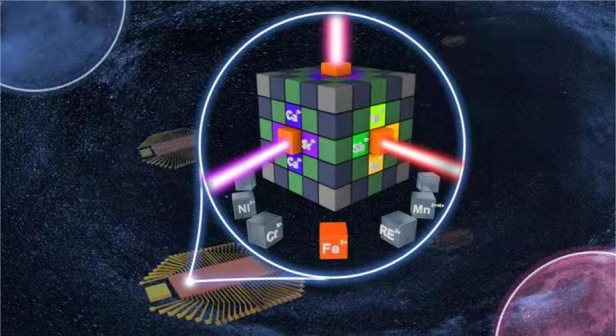

## Introduction

Near-infrared (NIR) light has been widely used in plant cultivation, night vision, food analysis, photovoltaics, and biomedicine^1–3^. With the increasing popularity of smart devices, it is necessary to develop portable NIR light sources. Traditional tungsten–halogen lamps are not compact, and NIR light-emitting diodes (LEDs) suffer from narrow spectral bandwidths^4,5^. Therefore, NIR-emitting phosphor-converted LEDs (pc-LEDs) have attracted great attention because of their small size, high efficiency, and tunable broadband emission^6,7^. However, exploitation of efficient and broadband NIR-emitting phosphors is one of the key obstacles encountered in the development of NIR-emitting pc-LEDs. The currently reported broadband NIR-emitting phosphors are mainly based on Cr^3+^ because it can usually produce broadband emission in the range 650–1200 nm when it is located in a weak octahedral coordination crystal field^8–11^. In addition, it can efficiently absorb at 460 nm due to a spin-allowed ^4^A_2g_ → ^4^T_1g_ transition, which matches well with the commercial blue LED chips. Moreover, substantial progress has been achieved in the tunable NIR luminescence of Cr^3+^-doped phosphor materials^12–14^. However, there is a potential risk of oxidation of Cr^3+^ to Cr^6+^ ^15,16^. On the one hand, mixed Cr^6+^ seriously affects the NIR luminescence efficiency^17^. On the other hand, this might increase the chromium toxicity of the phosphors, thereby limiting their practical applications in certain fields, particularly in long-term in vivo applications^18^. Therefore, there is an urgent requirement to find alternatives to the Cr^3+^ activator to achieve NIR emission. Recently, several studies have focused on Bi^3+^-, Eu^2+^-, and Mn^2+^-activated NIR-emitting phosphors^15,16,19^. Their emission wavelengths are near the deep-red light region, which has inferior penetration ability in biological tissues. Hence, efforts should be made to further tune the emission to a longer wavelength.

Another activator, Fe^3+^, is an essential element ion of the human body; moreover, it is non-toxic and can be regarded as a friendly dopant^20,21^. Thus, its optical properties are worth exploring. Although the luminescence of Fe^3+^ with intraconfigurational d–d transitions has been reported, the emission commonly occurs in the red and far-red light regions depending on the local environment of Fe^3+^ in the host materials^22–26^. Almost no Fe^3+^-doped phosphors exhibit NIR emission exceeding 800 nm, except for the recently reported CaAl_12_O_19_:Fe^3+^ (808 nm), SrAl_12_O_19_:Fe^3+^ (811 nm), and CaGa_2_O_4_:Fe^3+^ (809 nm)^27–29^. Typically, tetrahedrally coordinated Fe^3+^ ions emit in the 650−750 nm range, whereas octahedrally coordinated ones are expected to exhibit longer NIR emission wavelengths^30^. However, the d–d transitions of Fe^3+^ in the octahedral sites are more strictly restricted by the Laporte selection rule because of the higher symmetry of the octahedra^23,31^. Therefore, the achievement of long-wavelength NIR emission of Fe^3+^ with high luminescence efficiency is a challenging task.

The double-perovskite compounds generally show excellent optical properties, physical stability, and chemical stability^32^. NIR emission of Cr^3+^ has been obtained in the double-perovskite structures^6,33^. The double-perovskite hosts with typically octahedral sites have never been used for Fe^3+^ doping, the crystal structures of which can be flexibly modulated by cation substitution. Thus the tunable luminescence of Fe^3+^ can be expected. Furthermore, the octahedral In^3+^ sites are common for Cr^3+^ doping^7,34,35^. Given that Fe^3+^ and Cr^3+^ have the same valence state and similar ionic radii, an In-based double-perovskite Sr_2_InSbO_6_ was chosen as the initial host for Fe^3+^ doping in this work. Here, a series of Fe^3+^-activated Sr_2-*y*_Ca_*y*_(InSb)_1−*z*_Sn_2*z*_O_6_ NIR-emitting phosphors with uncommon long-wavelength NIR emission of Fe^3+^ were synthesized. Tunable emission from 885 to 935 and then up to 1005 nm were achieved by the premeditated cation substitution of Ca^2+^ for Sr^2+^ and further cosubstitution of [Sn^4+^–Sn^4+^] for [In^3+^–Sb^5+^]. The full-width at half maximum (FWHM) was broadened from 108 to 146 nm during this process. The complete introduction of Ca^2+^ significantly improved the luminescence efficiency, thereby reaching an ultra-high internal quantum efficiency (IQE) of 87% for Ca_2_InSbO_6_:Fe^3+^. The structure-related emission-tunable properties and the corresponding luminescence mechanism were analyzed. The feasibility of the as-synthesized phosphors in applications such as night vision, nondestructive biological monitoring, and NIR spectroscopy detection were also investigated.

## Results

### Crystal structure and phase identification

**Fig. 1:**
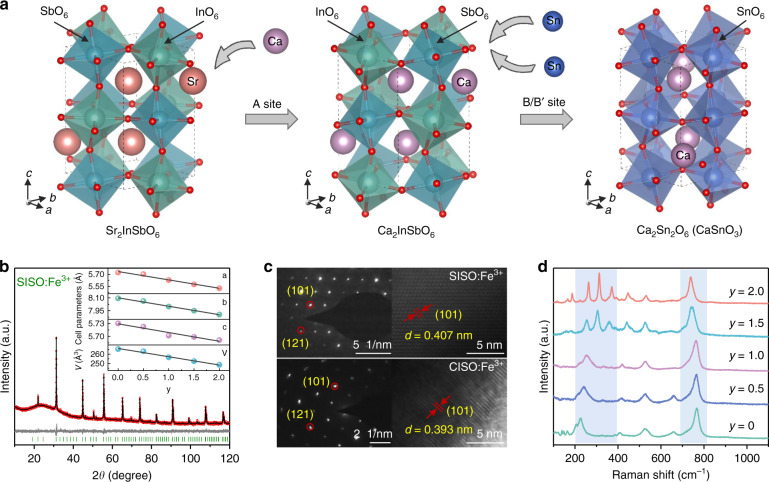
Structure characterizations of Sr_2−*y*_Ca_*y*_(InSb)_1−*z*_Sn_2*z*_O_6_:Fe^3+^. **a** Schematic illustration of the composition transformation from SISO to CISO and to CSO via cation substitution. **b** XRD Rietveld refinement of SISO:Fe^3+^. The inset shows the cell parameters and volume variations of Sr_2-*y*_Ca_*y*_InSbO_6_:Fe^3+^ (*y* = 0–2). **c** HRTEM images with the SAED patterns of SISO:Fe^3+^ and CISO:Fe^3+^. **d** Raman spectra of Sr_2-*y*_Ca_*y*_InSbO_6_:Fe^3+^ (*y* = 0–2)

As shown in Fig. 1a, Sr_2_InSbO_6_ (SISO) adopts a double-perovskite structure with the general formula A_2_BB’O_6_, which consists of alternately arranged [InO_6_] and [SbO_6_] octahedra with larger Sr cations occupying the voids between the octahedra. The cation substitution of Ca^2+^ for Sr^2+^ at A sites leads to composition transformation from SISO to Ca_2_InSbO_6_ (CISO), followed by the cosubstitution of [Sn^4+^–Sn^4+^] for [In^3+^–Sb^5+^] at B and B’ sites to obtain the CaSnO_3_ (CSO) phase. The detailed substitution process was revealed by the X-ray diffraction (XRD) patterns of Sr_2-*y*_Ca_*y*_InSbO_6_:Fe^3+^ (*y* = 0–2) and Ca_2_(InSb)_1-*z*_Sn_2*z*_O_6_:Fe^3+^ (*z* = 0–1) (Fig. S1). The Bragg reflections shifting toward higher angles indicates the lattice shrinkage, which is attributed to the smaller Ca^2+^ and [Sn^4+^–Sn^4+^] substituting Sr^2+^ and [In^3+^–Sb^5+^], respectively (Table S1). To verify the phase purity of the as-synthesized phosphors, the Rietveld refinement was performed, as shown in Figs. 1b, S2, and S3. The refined crystallographic parameters and main bond lengths are listed in Tables S2 and S3, respectively. As expected, the cell parameters (*a*, *b*, *c*) and volume (*V*) decreased with the introduction of Ca^2+^ and Sn^4+^ (Figs. 1b and S4), demonstrating the lattice shrinkage. These results also indicate the successful realization of the designed substitution. The elemental mappings of scanning electron microscopy (SEM) in Fig. S5 show the evenly distributed composition elements of SISO:Fe^3+^, CISO:Fe^3+^, and CSO:Fe^3+^. Figure 1c shows the high-resolution transmission electron microscopy (HRTEM) images of SISO:Fe^3+^ and CISO:Fe^3+^. The (101) and (121) crystal planes were differentiated in the selected area electron diffraction (SAED) patterns. The interplanar spacing value of the (101) plane was decreased from 4.07 Å to 3.93 Å, which further confirmed the lattice shrinkage after Ca^2+^ completely substituted Sr^2+^. Structural changes in Sr_2-*y*_Ca_*y*_InSbO_6_:Fe^3+^ were also reflected in the Raman spectra (Fig. 1d). The strong bands in the 200–350 cm^−1^ region are assigned to the stretching vibrations of Sr/Ca–O^36,37^. Peak broadening and splitting appeared after Ca^2+^ incorporation, indicating a reduced structural symmetry. Furthermore, the peak shifting to higher wavenumbers is associated with the shortened Sr/Ca–O bond lengths, as derived from the Rietveld refinements (Table S3). Another intense band (700–800 cm^−1^) can be attributed to the symmetric stretching of the [BO_6_] and [B’O_6_] octahedra^38^, which shifts to lower wavenumbers with Ca^2+^ substituting Sr^2+^. A similar phenomenon was found for A_2_NdSbO_6_ (*A* = Ba, Sr, Ca), which is attributed to the increase in the Sb–O bond length^39^. These structural changes lay the foundation for the luminescence tuning of Fe^3+^.

### Photoluminescence properties

**Fig. 2:**
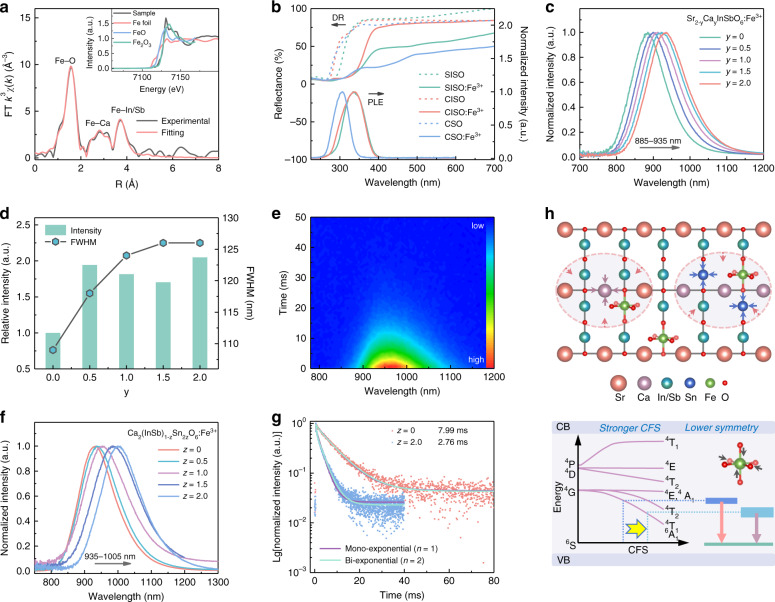
Photoluminescence properties of Sr_2-*y*_Ca_*y*_(InSb)_1−*z*_Sn_2*z*_O_6_:Fe^3+^. **a** Final fitting results of the Fourier-transformed Fe EXAFS spectra of Ca_2_In_0.88_SbO_6_:0.12Fe^3+^ in *R* space. The inset shows the normalized Fe K-edge XANES spectra and the reference compounds of Fe foil, FeO, and Fe_2_O_3_. **b** DR and PLE spectra of SISO:Fe^3+^, CISO:Fe^3+^, and CSO:Fe^3+^. **c** Normalized PL spectra of Sr_2-*y*_Ca_*y*_InSbO_6_:Fe^3+^ (*y* = 0–2). **d** PL intensity and FWHM of Sr_2-*y*_Ca_*y*_InSbO_6_:Fe^3+^ (*y* = 0–2). **e** TRPL spectra of CISO:Fe^3+^. **f** Normalized PL spectra of Ca_2_(InSb)_1-*z*_Sn_2*z*_O_6_:Fe^3+^ (*z* = 0–1). **g** Luminescence decay curves of CISO:Fe^3+^ and CSO:Fe^3+^. **h** Schematic diagram of the overall PL tuning mechanism

X-ray absorption fine structure (XAFS) was investigated to analyze the local coordination environment of Fe^3+^. As these hosts were isomorphic, only the coordination environment of Fe^3+^ in CISO was analyzed. In order to obtain the clear signal of Fe, Ca_2_In_0.88_SbO_6_:0.12Fe^3+^ with a larger Fe doping concentration was chosen to obtain the XAFS data, as shown in Fig. 2a. The Fe K-edge X-ray absorption near-edge structure (XANES) spectrum (the inset of Fig. 2a) of the sample is close to that of the standard Fe_2_O_3_, indicating that the valence state of iron in the sample is mainly trivalent (Fe^3+^). Divalent Fe^2+^ also coexists in the sample, the amount of which is determined to be 17.6% of total doped Fe by quantitative analysis of the XANES spectra. These Fe^2+^ ions could be transformed to Fe^3+^ ions by changing the reaction conditions such as sintering under O_2_ atmosphere. The fitting results of the Fourier-transformed extended X-ray absorption fine structure(EXAFS) spectrum are shown in Table S4. The first, second, and third coordination shells of Fe is O, Ca, and In/Sb, respectively. For the Fe–O shell, the coordination number (CN) is calculated to be 7.1 (±0.9), which is larger than the expected CN of 6. This should be attributed to the larger error of the CN obtained by EXAFS data fitting, which can generally reach 10% or even 20%. The average interionic distance of Fe–O is 2.04 Å, which is relatively consistent with the refinement result. Based on the diffuse reflection (DR) spectra, the band gap values of SISO, CISO, and CSO hosts were estimated to be 4.2, 4.5, and 4.6 eV, respectively (Fig. S6). A large band gap implies that there is sufficient energy gap to accommodate the energy levels of doped Fe^3+^. As indicated by the DR spectra in Fig. 2b, SISO:Fe^3+^, CISO:Fe^3+^, and CSO:Fe^3+^ exhibit significantly enhanced absorption in the 250–400 nm region as compared with the hosts, which is ascribed to the O^2−^–Fe^3+^ charge transfer (CT) transition. The photoluminescence excitation (PLE) spectra (Fig. 2b) of SISO:Fe^3+^, CISO:Fe^3+^, and CSO:Fe^3+^ are consistent with their corresponding DR spectra and show strong CT bands. The d–d transitions of Fe^3+^ can hardly be observed because of the spin and parity forbidden feature, the transition probability of which is highly dependent on the site symmetry^27^. Figure S7a shows the PLE spectra of SISO:Fe^3+^ and CISO:Fe^3+^ at 7 K. In addition to the dominant CT band, weak peaks assigned to the ^6^A_1_ (^6^S) → ^4^E (^4^D) (405 nm), ^6^A_1_ (^6^S) → ^4^T_2_ (^4^D) (465 nm), and ^6^A_1_ (^6^S) → ^4^T_2_ (^4^G) (580 nm) transitions of Fe^3+^ are also presented in the enlarged PLE spectra (Fig. S7b). It should be noted that these transitions of Fe^3+^ in CISO are more intense than those in SISO, suggesting the lower symmetry of the Fe^3+^ sites in CISO.

It was found that the hosts of this series of phosphors exhibited NIR emission with a certain intensity. Evidence suggests that such host luminescence occurs due to the unintentional Fe^3+^ impurity, which is explained in the Supporting Information according to the photoluminescence (PL) properties and ICP results (Figs. S8–S11 and Tables S5 and S6). Figure 2c shows PL spectra of Sr_2-*y*_Ca_*y*_InSbO_6_:Fe^3+^ (*y* = 0–2). Under an excitation wavelength of 340 nm, SISO:Fe^3+^ exhibited a broad NIR emission band centered at 885 nm, originating from the ^4^T_1_ (^4^G) → ^6^A_1_ (^6^S) transition of the Fe^3+^ ions in the octahedral sites. With the cation substitution of Ca^2+^ for Sr^2+^, the emission spectra showed a continuous red shift from 885 to 935 nm, accompanied by a broadened FWHM from 108 to 126 nm (1441 to 1477 cm^−1^). Meanwhile, the emission intensity was greatly increased, as shown in Fig. 2d. A more than 2-fold increase in the PL intensity of the Fe^3+^ ions was achieved when Ca^2+^ completely substituted Sr^2+^. The lattice shrinkage caused by smaller Ca^2+^ substituting larger Sr^2+^ can lead to a stronger crystal field strength (CFS) around Fe^3+^ according to the relationship of *D*_q_ vs. *R*^−5^ ^12^, thereby reducing the energy difference between its ^4^T_1_ (^4^G) excited state and ^6^A_1_ (^6^S) ground state. Thus, a normal red shift in the PL spectra was observed. As mentioned earlier, Ca^2+^ incorporation also lowers the site symmetry of Fe^3+^, which can facilitate the breaking of the forbidden transition of Fe^3+^ and improve the luminescence. Moreover, the lower structural symmetry can introduce an uneven crystal field that results in the formation of ^4^T_1_ (^4^G) sub-levels, thereby broadening the FWHM of the PL spectra. The PL decay curves of Sr_2-*y*_Ca_*y*_InSbO_6_:Fe^3+^ are shown in Fig. S12, which can be well fitted by the bi-exponential function^15^:

1 $$I(t) = A_1{{{\mathrm{exp}}}}\left( { - \frac{t}{{\tau _1}}} \right) + A_2{{{\mathrm{exp}}}}\left( { - \frac{t}{{\tau _2}}} \right)$$

2 $$\tau ^ \ast = \frac{{A_1\tau _1^2 + A_2\tau _2^2}}{{A_1\tau _1 + A_2\tau _2}}$$

where *I*(*t*) is the luminescence intensity at time *t*, *A*_1_, and *A*_2_ are fitted constants, and *τ*_1_ and *τ*_2_ are the rapid and slow lifetimes of exponential components, respectively. The average lifetime (τ*) values for *y* = 0, 1, and 2 were calculated to be 8.32, 7.34, and 7.99 ms (Table S7), respectively. The relatively long decay times are related to the forbidden characteristic of the ^4^T_1_ (^4^G) → ^6^A_1_ (^6^S) transition. The time-resolved photoluminescence (TRPL) spectra (Fig. 2e) of CISO:Fe^3+^ showed that only one Fe^3+^ luminescent center contributed to the luminescence. Figure S13a shows the low temperature PL spectrum of CISO:Fe^3+^ measured at 7 K, which shows a slight asymmetry in the emission band. An asymmetric spectral profile generally means more than one luminescent center. However, it can be seen that the two PLE spectra in Fig. S13b monitored at 930 and 1000 nm almost completely overlap. Moreover, the luminescence decay curves monitored at 930 and 1000 nm are nearly consistent (Fig. S13c). These results prove one luminescent center in CISO:Fe^3+^. To further verify this point, the low temperature TRPL spectra of CISO:Fe^3+^ were also measured, as shown in Fig. S13d. It is seen that the PL intensity decreases synchronously as the decay time is prolonged, and no visible change in spectral shape can be observed. Therefore, it is reasonable to ascribe this broadband emission to one Fe^3+^ luminescence center. Considering the valence state and ion radius, it is speculated that the Fe^3+^ ions occupy the octahedral In^3+^ sites in CISO:Fe^3+^.

The aliovalent substitution of [Sn^4+^–Sn^4+^] for [In^3+^–Sb^5+^] was further performed to tune the NIR emission, as shown in Fig. 2f. The PL spectra of Ca_2_(InSb)_1-*z*_Sn_2*z*_O_6_:Fe^3+^ (*z* = 0–1) exhibited a red shift from 935 to 1005 nm and a broadened FWHM from 126 to 146 nm (1477 to 1485 cm^−1^), which can be attributed to the previously discussed strengthened CFS and formation of energy sub-levels, respectively. However, the PL intensity decreased dramatically with the incorporation of Sn^4+^ (Fig. S14). The TRPL spectra also revealed one luminescent center in CSO:Fe^3+^ (Fig. S15), indicating the occupation of octahedral Sn^4+^ sites by the Fe^3+^ ions. Unlike the alternate arrangement of [InO_6_] and [SbO_6_] octahedra in SISO and CISO (Fig. 1a), the adjacent arrangement of the [SnO_6_] octahedra in CSO indicates a shorter distance between the Fe^3+^ luminescent centers, which can lead to a significant concentration quenching effect that reduces the PL intensity. The concentration quenching is caused by the energy transfer among Fe^3+^ ions. To analyze the energy transfer mechanism between Fe^3+^ ions in the CSO host, the critical distance (*R*_c_) is estimated by the following formula^40^:

3 $$R_{{{\mathrm{c}}}} \approx 2\left( {\frac{{3V}}{{4\pi x_{{{\mathrm{c}}}}N}}} \right)^{\frac{1}{3}}$$

where *x*_c_ is the critical concentration of Fe^3+^ ions, *N* is the total sites of Fe^3+^ per unit cell, *V* is the volume of the unit cell. Accordingly, the *R*_c_ value was determined to be about 33.96 Å, which is much larger than 5 Å. It is reasonable to attribute the energy transfer mechanism to the electric multipolar interaction rather than the exchange interaction. The energy transfer mechanism of electric multipolar interactions can be determined by the value of *θ* from the following formula^41^:

4 $${{{\mathrm{log}}}}\left( {\frac{I}{x}} \right) = A - \frac{\theta }{3}{{{\mathrm{log}}}}x$$

where *x* and *I* represent the doping concentration and the corresponding emission intensity, respectively. *A* is a constant, and *θ* values of 6, 8, 10 correspond to dipole–dipole, dipole–quadrupole, and quadrupole–quadrupole interactions, respectively. As shown in Fig. S16, there is a good linear relationship between log (*I*/*x*) and log (*x*), and the *θ* value is finally determined to be 4.6, which is close to 6. Thus, the concentration quenching mechanism is attributed to the dipole–dipole interaction.

Figure 2g shows the PL decay curves of CISO:Fe^3+^ and CSO:Fe^3+^. It can be seen that the decay curve of CISO:Fe^3+^ is well fitted by the mono-exponential function, whereas that of CSO:Fe^3+^ deviates from the mono-exponential fitting and is fitted by the bi-exponential function, indicating an additional energy decay path such as defect-induced nonradiative relaxation process^42^. In CaSnO_3_, there is a structural possibility for an enhanced exchange interaction between the Fe^3+^ ions. This should also result in a red-shifted emission besides the stronger CFS, as was indeed observed in Fig. 2f. Due to the exchange interaction, the originally spin quartet excited state of Fe^3+^ acquires spin sextet character, which should also decrease its decay time as is observed. Another explanation could be the requirement for charge compensation upon substitution of Sn^4+^ by Fe^3+^, which could lead to a close charge-compensating defect that distorts the site and could thus, also lower the decay time. Accordingly, the lifetime was shortened from 7.99 to 2.76 ms with the complete introduction of Sn^4+^ (Table S8). Figure 2h shows a schematic diagram of the overall PL tuning mechanism. As discussed above, the designed substitution of Ca^2+^ for Sr^2+^ and cosubstitution of [Sn^4+^–Sn^4+^] for [In^3+^–Sb^5+^] result in a stronger CFS and lower site symmetry, which are responsible for the observed emission red shift and broadening of the Fe^3+^ luminescence. The temperature-dependent luminescent properties were investigated, and the corresponding spectra (Figs. S17–19) are depicted in the Supporting Information. At 398 K, the integrated PL intensity decreased to 44%, 36%, and 24% of the initial intensity at 298 K for SISO:Fe^3+^, CISO:Fe^3+^, and CSO:Fe^3+^, respectively. The PL thermal stability requires further improvement for practical applications.

Quantum efficiency is an important parameter for evaluating the performance of phosphors. The IQEs of SISO:Fe^3+^ and CISO:Fe^3+^ were measured to be 48% and 87%, respectively (Fig. S20a, b). The absorption efficiency and external quantum efficiency (EQE) were estimated and presented in Supporting Information. The EQEs of SISO:Fe^3+^ and CISO:Fe^3+^ were estimated to be 35% and 68%, respectively. For reference, we synthesized the recently reported NaScGe_2_O_6_:Cr^3+^ and La_3_Ga_5_GeO_14_:Cr^3+^ phosphors with optimal doping content following relevant literature (Fig. S21)^10,43^. As shown in Fig. S22, the peak profiles and peak positions of the self-prepared samples of NaScGe_2_O_6_:Cr^3+^ and La_3_Ga_5_GeO_14_:Cr^3+^ are basically consistent with the reported ones. Moreover, their IQEs were measured to be 44% and 27% (Fig. S20c, d), respectively, which are close to those reported in the literature (Table 1), confirming the reliability of the measured IQEs in this work. The comparison of PL intensities of the as-synthesized phosphors in Fig. S22 agrees with that of the IQE results. Table 1 lists the IQEs of some Eu^2+^-, Mn^2+^-, and Cr^3+^-activated NIR-emitting phosphors. As the IQE for Fe^3+^ luminescence has been rarely reported, no Fe^3+^-doped NIR-emitting phosphors is mentioned in Table 1. It is shown that the IQE of CISO:Fe^3+^ is higher than those of K_3_LuSi_2_O_7_:Eu^2+^, MgAl_2_O_4_:Mn^2+^, and most Cr-activated NIR-emitting phosphors with peak wavelengths longer than 800 nm. Such a high IQE with simultaneous long-wavelength NIR emission (>900 nm) is rare in broadband NIR-emitting phosphors. Even the lower IQE of SISO:Fe^3+^ is comparable to that of Cr-activated phosphors with similar wavelengths. These facts demonstrate that Fe^3+^ is a promising candidate activator for highly efficient NIR emission.

**Table 1:** IQEs of some Eu^2+^-, Mn^2+^-, and Cr^3+^-activated NIR-emitting phosphors

Phosphor	*λ*_ex_ (nm)	*λ*_em_ (nm)	IQE (%)	Ref.
K_3_LuSi_2_O_7_:Eu^2+^	460	740	15	^15^
MgAl_2_O_4_:Mn^2+^	450	825	53	^19^
ScBO_3_:Cr^3+^	450	800	65	^44^
Ca_3_Y_2_Ge_3_O_12_:Cr^3+^	460	800	81	^45^
La_3_Sc_2_Ga_3_O_12_:Cr^3+^	480	818	35	^46^
Ga_1.6_In_0.4_O_3_:Cr^3+^	450	820	87.9	^34^
La_2_MgZrO_6_:Cr^3+^	460	825	58	^6^
K_2_Ga_2_Sn_6_O_16_:Cr^3+^	450	830	48	^47^
LiInSi_2_O_6_:Cr^3+^	460	840	75	^35^
Sr_9_Ga_0.2_(PO_4_)_7_:0.8Cr^3+^	450	850	66.3	^5^
LaSc_3_B_4_O_12_:Cr^3+^	460	871	23.29	^48^
LiScP_2_O_7_:Cr^3+^	470	880	38	^49^
NaScGe_2_O_6_:Cr^3+^	490	895	40.22	^10^
NaScGe_2_O_6_:Cr^3+^	490	888	44	This work
Mg_3_Ga_2_GeO_8_:Cr^3+^	425	915	35	^50^
La_3_Ga_5_GeO_14_:Cr^3+^	442	750, 920	20	^43^
La_3_Ga_5_GeO_14_:Cr^3+^	440	747, 920	27	This work
Mg_2_GeO_4_:Cr^3+^	470	940	48.19	^13^
LiIn_2_SbO_6_:Cr^3+^	492	970	7	^7^
Cs_2_AgInCl_6_:Cr^3+^	760	1010	22.03	^33^
Sr_2_InSbO_6_:Fe^3+^	340	885	48	This work
Ca_2_InSbO_6_:Fe^3+^	340	935	87	This work

### LED packages and applications

**Fig. 3:**
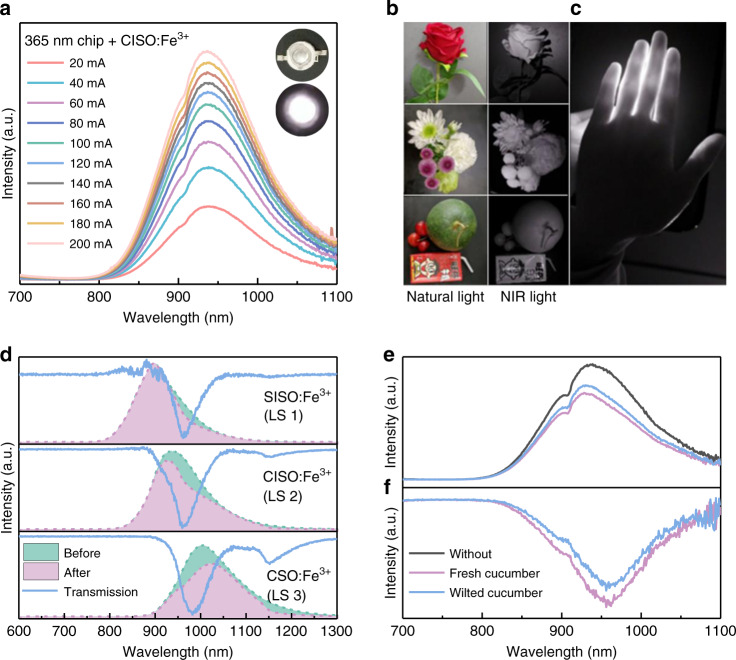
NIR applications based on pc-LEDs. **a** Emission spectra of the pc-LED fabricated by the CISO:Fe^3+^ phosphor. **b** Photographs captured under natural light and NIR light. **c** Photograph of the NIR light penetrating fingers. **d** PL spectra of the phosphor NIR light before and after penetrating water, and the corresponding calculated transmission spectra of water. **e** Emission spectra of the as-fabricated optimal pc-LED with and without cucumbers in the integrating sphere. **f** Absorption spectra of cucumbers

NIR-emitting pc-LEDs were manufactured to evaluate the application potential of the synthesized phosphors. Their driving current-dependent PL spectra are shown in Figs. 3a and S23, and the corresponding NIR output power is given in Fig. S24. A maximum NIR output power of 0.83 mW at 200 mA was obtained in the pc-LED fabricated by CISO:Fe^3+^, which can be further optimized. Figure 3b presents photographs of some objects under natural light and the optimal NIR-emitting pc-LED light, indicating the potential application of CISO:Fe^3+^ in night vision. In addition, the NIR-emitting pc-LED light can penetrate fingers (Fig. 3c), which provides the possibility of using CISO:Fe^3+^ for nondestructive examination of biological tissues.

Other potential fields, such as NIR spectroscopy analysis, can be expected for the developed emission-tunable NIR-emitting phosphors. Here, the phosphors are regarded as light sources (LS), the emission of which can be selectively absorbed by the functional groups. Figure 3d shows the PL spectra (shadow-filled) of SISO:Fe^3+^ (LS 1), CISO:Fe^3+^ (LS 2), and CSO:Fe^3+^ (LS 3) before and after penetrating water. The corresponding calculated transmission spectra of water are plotted as solid lines in Fig. 3d. It can be observed that all three light sources sensitively detect the 975 nm absorption signal of water. As for the 1150 nm absorption peak, LS 1 and LS 2 are almost undetectable, whereas LS 3 can recognize a relatively obvious signal. This result is due to the different effective spectral regions of the light sources. Figure S25 shows comparison of the emission spectra of the as-synthesized SISO:Fe^3+^, CISO:Fe^3+^, CSO:Fe^3+^ phosphors and 940 nm chip. It is observed that the as-synthesized phosphors show much broader bandwidth than that of commercial 940 nm chip. Moreover, the emission wavelength of the phosphors is tunable. Merging these emission-tunable phosphors into one LED can enlarge the spectrum region to cover more information for NIR spectroscopic analysis, which is of great importance for emission tuning and broadening of the NIR-emitting phosphors.

To demonstrate the application potential of the as-fabricated optimal NIR pc-LED in food analysis, cucumbers with varying freshness levels were selected for detection. Figure 3e shows the emission spectra of the NIR pc-LED with and without the cucumbers in the integrating sphere. It can be seen that the cucumbers show broadband absorption in the 850–1100 nm range owing to the water content absorption (Fig. 3f). Moreover, the fresh cucumber that contained more water exhibited more obvious NIR absorption. These results indicate the potential application of the NIR pc-LEDs in nondestructive food analysis based on the NIR spectroscopic analysis.

## Discussion

In summary, a series of Fe^3+^-activated Sr_2-*y*_Ca_*y*_(InSb)_1−*z*_Sn_2*z*_O_6_ broadband NIR-emitting phosphors were designed and successfully synthesized. Under 340 nm excitation, SISO:Fe^3+^ showed a broad NIR emission band centered at 885 nm. Controllable emission tuning from 885 to 935 nm with significantly enhanced PL intensity was achieved by cation substitution of Ca^2+^ for Sr^2+^. The subsequent cation cosubstitution of [Sn^4+^–Sn^4+^] for [In^3+^–Sb^5+^] further tuned the emission from 935 to 1005 nm. The continuous emission red shift is a result of the strengthened CFS induced by lattice shrinkage. Moreover, the complete introduction of Ca^2+^ and Sn^4+^ broadened the FWHM from 108 to 146 nm. The SISO:Fe^3+^ and CISO:Fe^3+^ phosphors exhibited high IQEs of 48% and 87%, respectively, and EQEs of 35% and 68%, respectively, indicating the potential of Fe^3+^ activator in obtaining highly efficient NIR emission. Further, the as-fabricated NIR pc-LEDs showed potential applications in night vision, nondestructive biological monitoring, and NIR spectroscopy detection. This work provides new insights into the luminescence of Fe^3+^, which opens up a new avenue for the development of highly efficient broadband NIR-emitting phosphor materials.

## Materials and methods

### Materials synthesis

Sr_2-*y*_Ca_*y*_(InSb)_1-*z*_Sn_2*z*_O_6_:*x*Fe^3+^ (*x* = 0–0.03, *y* = 0–2, *z* = 0–1) phosphors were synthetized *via* a high-temperature solid-state reaction process. Strontium carbonate (SrCO_3_, S. P.), calcium carbonate (CaCO_3_, A. R.), indium oxide (In_2_O_3_, 99.99%), antimony trioxide (Sb_2_O_3_, 99.99%), and ferric sesquioxide (Fe_2_O_3_, 99.99%) were obtained from Aladdin Reagent Co., Ltd. Tin oxide (SnO_2_, S. P.) was acquired from Sinopharm Chemical Reagent Co., Ltd. The raw materials were stoichiometrically weighed and thoroughly ground in an agate mortar for 20 min. The precursors were transferred to alumina crucibles and sintered at 1573 K for 6 h in a box furnace. The resulting products were slowly cooled down to room temperature and ground again.

La_3_Ga_4.95_GeO_14_:0.05Cr^3+^ and NaScGe_2_O_6_:0.03Cr^3+^ phosphors for IQE comparison were synthesized according to the literature^10,43^. Lanthanum oxide (La_2_O_3_, 99.999%), scandium oxide (Sc_2_O_3_, 99.999%), gallium oxide (Ga_2_O_3_, 99.99%), sodium carbonate (Na_2_CO_3_, A. R.), germanium oxide (GeO_2_, 99.99%), and chromium oxide (Cr_2_O_3_, 99.99%) were obtained from Aladdin Reagent Co., Ltd. The stoichiometric amounts of La_2_O_3_, Ga_2_O_3_, GeO_2_, and Cr_2_O_3_ were weighed and thoroughly ground in an agate mortar for 20 min, and the precursors were transferred to alumina crucibles and sintered at 1523 K for 5 h in air atmosphere to obtain the La_3_Ga_4.95_GeO_14_:0.05Cr^3+^ phosphor. The stoichiometric amounts of Na_2_CO_3_, Sc_2_O_3_, GeO_2_, and Cr_2_O_3_ were weighed and thoroughly ground in an agate mortar for 20 min, and the precursors were transferred to alumina crucibles and sintered at 1473 K for 5 h in air atmosphere to obtain the NaScGe_2_O_6_:0.03Cr^3+^ phosphor.

### LED fabrication

The as-prepared NIR phosphors were thoroughly mixed with silicone resins A and B (A:B=1:1). The mixtures were then coated on the 365 and 310 nm chips, and cured at 150 °C for 1 h to obtain the final LED devices.

### Characterization

The X-ray diffraction (XRD) patterns of the as-synthesized phosphors were measured on a Bruker D8 ADVANCE powder diffractometer (Cu Kα radiation, *λ* = 1.54 Å) within the 2*θ* range 10−70°. XRD Rietveld refinements were conducted using the TOPAS 4.2. The field-emission scanning electron microscope (FE-SEM, S-4800, Hitachi) equipped with an Energy Dispersive Spectrometer (EDS) was used to obtain the elemental compositions. The high-resolution transmission electron microscopes (HRTEM) images were acquired using a FEI Tecnai G2 S-Twin. Raman spectra were recorded on a Raman spectrometer (Model T64000, Horiba JobinYvon, France) with a 512 nm laser. The X-ray absorption experiments were carried out at the XAS station (BL14W1) of the Shanghai Synchrotron Radiation Facility. The electron storage ring was operated at 3.5 GeV. Si (311) double-crystal was used as the monochromator, and the data was collected using solid-state detector under ambient conditions. The beam size was limited by the horizontal and vertical slits with the area of 1 × 4 mm^2^ during XAS measurements. The diffuse reflection (DR) spectra were recorded on a UV−vis−NIR spectrophotometer (UV-3600 plus, Shimadzu, Japan). The photoluminescence excitation (PLE) and photoluminescence (PL) spectra were measured by an Edinburgh Instruments FLSP-920 fluorescence spectrometer with a 450 W xenon lamp as excitation source, and an R5509-72 photomultiplier (PMT) as a light detector. The R5509-72 PMT is a nitrogen-cooled NIR-sensitive PMT, and the cooling temperature is −85 °C. The PL decay curves and time-resolved photoluminescence (TRPL) spectra were also measured by an Edinburgh Instruments FLSP-920 fluorescence spectrometer with a μF2 lamp as excitation source. The temperature-dependent PL spectra and decay curves were also measured by Edinburgh Instruments FLSP-920 fluorescence spectrometers equipped with a temperature controller. The IQEs were measured by an Edinburgh Instruments FLS-1000 equipped with an optical integrating sphere. Xenon lamp was the excitation source, and R5509-72 PMT was used as a light detector. The element content was determined by the inductive Coupled Plasma-atomic emission (ICP-AES) spectrometer (Agilent 7800). The emission spectra of the as-fabricated phosphor-converted light-emitting diodes (pc-LEDs) are mearsured on the HAAS 2000 photoelectric measuring system from EVERFINE. The photographs in the application of NIR pc-LEDs are taken by a NIR and a visible camera.

## Supplementary information

Highly efficient Fe3+-doped A2BB’O6 (A = Sr2+, Ca2+; B, B’ = In3+, Sb5+, Sn4+) broadband near-infrared-emitting phosphors for spectroscopic analysis

文章保密与发表审查单
