# Supplementary material for: Highly efficient Fe3+-doped A2BB′O6 (A = Sr2+, Ca2+; B, B′ = In3+, Sb5+, Sn4+) broadband near-infrared-emitting phosphors for spectroscopic analysis
Source: Light Sci Appl. 2022 Apr 27;11:112. doi: 10.1038/s41377-022-00803-x (PMC9046267; doi:10.1038/s41377-022-00803-x)
Supplement: Supplementary file 1 — Highly efficient Fe3+-doped A2BB’O6 (A = Sr2+, Ca2+; B, B’ = In3+, Sb5+, Sn4+) broadband near-infrared-emitting phosphors for spectroscopic analysis [file 41377_2022_803_MOESM1_ESM.docx]

Supplementary Information for

**Highly efficient Fe^3+^-doped A_2_BB’O_6_ (A = Sr^2+^, Ca^2+^; B, B’ = In^3+^, Sb^5+^, Sn^4+^) broadband near-infrared-emitting phosphors for spectroscopic analysis**

Dongjie Liu,^1,3^ Guogang Li,^2,4,^* Peipei Dang,^1,3^ Qianqian Zhang,^1,3^ Yi Wei,^2^ Lei Qiu,^2^ Maxim S. Molokeev^5,6,7^ Hongzhou Lian,^1^ Mengmeng Shang,^8^ and Jun Lin^1,3,^*

^1^ State Key Laboratory of Rare Earth Resource Utilization, Changchun Institute of Applied Chemistry, Chinese Academy of Sciences, Changchun 130022, China. E-mail: jlin@ciac.ac.cn

^2^ Faculty of Materials Science and Chemistry, China University of Geosciences, Wuhan 430074, China. E-mail: ggli@cug.edu.cn

^3^ University of Science and Technology of China, Hefei 230026, China

^4^ Zhejiang Institute, China University of Geosciences, Hangzhou, 311305, China

^5^ Laboratory of Crystal Physics, Kirensky Institute of Physics, Federal Research Center KSC SB RAS, Krasnoyarsk 660036, Russia

^6^ Institute of Engineering Physics and Radioelectronics, Siberian Federal University, Krasnoyarsk 660041, Russia

^7^ Research and Development Department, Kemerovo State University, Kemerovo 650000, Russia

^8^ School of Material Science and Engineering, Shandong University, Jinan 266071, China

**Fig. S1** XRD patterns of **a** Sr_2-_*_y_*Ca*_y_*InSbO_6_:Fe^3+^ (*y* = 0–2) and **b** Ca_2_(InSb)_1-_*_z_*Sn_2_*_z_*O_6_:Fe^3+^ (*z* = 0–1).

All XRD peaks of SISO:Fe^3+^ and CISO:Fe^3+^ were indexed by monoclinic cell (*P*2_1_/*n*) with parameters close to Sr_2_InSbO_6_ and Ca_2_InSbO_6_.^1^ Therefore, the two structures were taken as starting models for Rietveld refinement. There are two octahedral sites in the asymmetric part of the unit cell which were occupied by In^3+^ and Sb^5+^ ions. Rietveld refinement is performed under the constraints of In/Sb cation ordering. The ratio In/Sb was refined in each site taking into consideration that sum of all ions in site equal to 1. Refinements were stable and gave low *R*-factors (Fig. S2 and Table S2).

In order to simplify the refinement process of Sr_2-_*_y_*Ca*_y_*InSbO_6_:Fe^3+^, we reduced the numbers of refined parameters, that is, the two octahedral sites were occupied by In^3+^ and Sb^5+^ ions with fixed occupancies according to suggested chemical formula. As a result, we got much higher symmetry and were able to fit all patterns by *Pnma* model with very small amount of parameters. Refinements were still stable and gave low *R*-factors (Fig. S3 and Table S2). It is found that the refined cell parameters *a*, *b*, *c*, and *V* by *Pnma* model are very close to those obtained by *P*2_1_*/n* model. The difference is that the parameter *β* obtained from *P*2_1_*/n* model slightly deviates from 90°. For CaSnO_3_:Fe^3+^, all peaks were indexed by orthorhombic cell (*Pnma*) with parameters close to CaSnO_3_.^2^ Therefore, this structure was taken as starting model for Rietveld refinement of Ca_2_(InSb)_1-_*_z_*Sn_2_*_z_*O_6_:Fe^3+^. Model refinements for all compounds were stable and gave low *R*-factors.

**Fig. S2** XRD Rietveld refinement of SISO:Fe^3+^ and CISO:Fe^3+^ following *P*2_1_/*n* model.

**Fig. S3** XRD Rietveld refinement of Sr_2-_*_y_*Ca*_y_*(InSb)_1-_*_z_*Sn_2_*_z_*O_6_:Fe^3+^ (*y* = 0–2, *z* = 0–1) following *Pnma* model.

**Table S1** Ion radii and coordination numbers (CN) of Sr^2+^, Ca^2+^, In^3+^, Sb^5+^, Sn^4+^, and Fe^3+^.

| Ion | CN | Ionic Radius |
| --- | --- | --- |
| Sr^2+^ | 12 | 1.44 Å |
| Ca^2+^ | 12 | 1.34 Å |
| In^3+^ | 6 | 0.8 Å |
| Sb^5+^ | 6 | 0.6 Å |
| Sn^4+^ | 6 | 0.69 Å |
| Fe^3+^ | 6 | 0.645 Å |

**Table S2** Main parameters of processing and refinement results of Sr_2-_*_y_*Ca*_y_*(InSb)_1-_*_z_*Sn_2_*_z_*O_6_:Fe^3+^ (*y* = 0–2, *z* = 0–1).

| *y* | *z* | Space Group | Cell parameters (Å ),  Cell Volume (Å^3^) | *R*_wp_, *R*_p_, *R*_B_, *χ*^2^ |
| --- | --- | --- | --- | --- |
| 0 | 0 | *Pnma* | *a* = 5.7230 (2),  *b* = 8.0948 (3),  *c* = 5.7304 (3),  *V* = 265.473 (19) | 5.98, 4.49, 1.28, 1.20 |
|  |  |  |  |  |
|  |  | *P*2_1_*/n* | *a* = 5.72686 (18), | 5.95, 4.52, 1.00, 1.27 |
|  |  |  | *b* = 5.72169 (19), |  |
|  |  |  | *c* = 8.0972 (3), |  |
|  |  |  | *V* = 265.324 (15), |  |
|  |  |  | *β = 90.042 (2)* |  |
|  |  |  |  |  |
| 0.5 | 0 | *Pnma* | *a* = 5.7018 (12),  *b* = 8.0630 (9),  *c* = 5.7227 (9),  *V* = 263,09 (7) | 5,69, 4,42, 1.85, 1.11 |
|  |  |  |  |  |
| 1 | 0 | *Pnma* | *a* = 5.6278 (7),  *b* = 7.9984 (12),  *c* = 5.7019 (6),  *V* = 256.66 (6) | 5.93, 4.50, 1.00, 1.18 |
|  |  |  |  |  |
| 1.5 | 0 | *Pnma* | *a* = 5.5747 (3),  *b* = 7.9501 (4),  *c* = 5.6987 (3),  *V* = 252.56 (2) | 6.56, 4.96, 1.65, 1.26 |
|  |  |  |  |  |
| 2 | 0 | *Pnma* | *a* = 5.52650 (12),  *b* = 7.90516 (17),  *c* = 5.69148 (13),  *V* = 248.649 (10) | 7.11, 5.33, 2.06, 1.35 |
|  |  |  |  |  |
|  |  | *P*2_1_*/n* | *a* = 5.52484 (12), | 5.68, 4.29, 1.05, 1.15 |
|  |  |  | *b* = 5.68902 (12), |  |
|  |  |  | *c* = 7.90250 (17), |  |
|  |  |  | *V* = 248.383 (9), |  |
|  |  |  | *β = 89.9184 (14)* |  |
|  |  |  |  |  |
| 2 | 0.25 | *Pnma* | *a* = 5.5283 (2),  *b* = 7.9019 (3),  *c* = 5.6761 (3),  *V* = 247.957 (19) | 6.36, 4.84, 1.86, 1.18 |
|  |  |  |  |  |
| 2 | 0.5 | *Pnma* | *a* = 5.5262 (2),  *b* = 7.8972 (4),  *c* = 5.6670 (3),  *V* = 247.32 (2) | 7.28, 5.54, 2.67, 1.34 |
|  |  |  |  |  |
| 2 | 0.75 | *Pnma* | *a* = 5.5215 (3),  *b* = 7.8892 (3),  *c* = 5.6599 (3),  *V* = 246.548 (19) | 6.89, 5.17, 2.52, 1.30 |
|  |  |  |  |  |
| 2 | 1 | *Pnma* | *a* = 5.51776 (18),  *b* = 7.8838 (2),  *c* = 5.65888 (19),  *V* = 246.166 (14) | 7.34, 5.51, 2.22, 1.37 |

**Table S3** Main bond lengths (Å) of Sr_2-_*_y_*Ca*_y_*(InSb)_1-_*_z_*Sn_2_*_z_*O_6_:Fe^3+^ (*y* = 0–2, *z* = 0–1).

| *y* = 0, *z* = 0 | | | |
| --- | --- | --- | --- |
| Sr—O1 (×2) | 2.88 (4) | (In/Sb)—O1 (×2) | 2.10 (4) |
| Sr—O1^i^ (×2) | 2.79 (3) | (In/Sb)—O1^iv^ (×2) | 2.06 (5) |
| Sr—O1^ii^ (×2) | 2.47 (3) | (In/Sb)—O2^iv^ (×2) | 2.051 (12) |
| Sr—O1 (×2) | 3.40 (4) | Average | 2.070 |
| Sr—O2 | 2.76 (4) |  |  |
| Sr—O2^iii^ | 2.56 (8) |  |  |
| Sr—O2 | 3.00 (4) |  |  |
| Sr—O2 | 3.18 (8) |  |  |
| Average | 2.882 |  |  |
| *y* = 0.5, *z* = 0 | | | |
| (Sr/Ca)—O1 (×2) | 2.90 (2) | (In/Sb)—O1(×2) | 2.18 (2) |
| (Sr/Ca)—O1^i^ (×2) | 2.785 (15) | (In/Sb)—O1^iv^ (×2) | 2.00 (2) |
| (Sr/Ca)—O1^ii^ (×2) | 2.372 (18) | (In/Sb)—O2^iv^ (×2) | 2.038 (4) |
| (Sr/Ca)—O1 (×2) | 3.466 (19) | Average | 2.073 |
| (Sr/Ca)—O2 | 2.70 (4) |  |  |
| (Sr/Ca)—O2^iii^ | 2.60 (3) |  |  |
| (Sr/Ca)—O2 | 3.04 (5) |  |  |
| (Sr/Ca)—O2 | 3.13 (4) |  |  |
| Average | 2.876 |  |  |
| *y* = 1, *z* = 0 | | | |
| (Sr/Ca)—O1 (×2) | 2.76 (4) | (In/Sb)—O1 (×2) | 2.02 (3) |
| (Sr/Ca)—O1^i^ (×2) | 2.79 (4) | (In/Sb)—O1^v^ (×2) | 2.04 (3) |
| (Sr/Ca)—O1^ii^ (×2) | 2.56 (4) | (In/Sb)—O2^v^ (×2) | 2.108 (12) |
| (Sr/Ca)—O1 (×2) | 3.26 (5) | Average | 2.056 |
| (Sr/Ca)—O2^iii^ | 2.38 (4) |  |  |
| (Sr/Ca)—O2^iv^ | 2.71 (3) |  |  |
| (Sr/Ca)—O2 | 3.06 (3) |  |  |
| (Sr/Ca)—O2 | 3.30 (5) |  |  |
| Average | 2.849 |  |  |
| *y* = 1.5, *z* = 0 | | | |
| (Sr/Ca)—O1 (×2) | 2.795 (16) | (In/Sb)—O1 (×2) | 2.029 (15) |
| (Sr/Ca)—O1^i^ (×2) | 2.789 (16) | (In/Sb)—O1^iii^ (×2) | 2.078 (13) |
| (Sr/Ca)—O1^ii^ (×2) | 2.310 (16) | (In/Sb)—O2^iii^ (×2) | 2.074 (5) |
| (Sr/Ca)—O1 (×2) | 3.452 (16) | Average | 2.060 |
| (Sr/Ca)—O2 | 2.097 (19) |  |  |
| (Sr/Ca)—O2^ii^ | 2.79 (3) |  |  |
| (Sr/Ca)—O2 | 3.08 (3) |  |  |
| (Sr/Ca)—O2 | 3.494 (19) |  |  |
| Average | 2.846 |  |  |
| *y* = 2, *z* = 0 | | | |
| Ca—O1^i^ (×2) | 2.659 (12) | (In/Sb)—O1 (×2) | 1.995 (11) |
| Ca—O1^ii^ (×2) | 2.461 (13) | (In/Sb)—O1^v^ (×2) | 2.110 (11) |
| Ca—O1 (×2) | 3.042 (13) | (In/Sb)—O2^v^ (×2) | 2.052 (3) |
| Ca—O1 (×2) | 3.231 (13) | Average | 2.052 |
| Ca—O2^iii^ | 2.308 (15) |  |  |
| Ca—O2^iv^ | 2.54 (2) |  |  |
| Ca—O2 | 3.23 (3) |  |  |
| Ca—O2 | 3.235 (16) |  |  |
| Average | 2.842 |  |  |
| *y* = 2, *z* = 0.25 | | | |
| Ca—O1^i^ (×2) | 2.656 (15) | (In/Sb/Sn)—O1 (×2) | 2.019 (15) |
| Ca—O1^ii^ (×2) | 2.461 (15) | (In/Sb/Sn)—O1^iv^ (×2) | 2.078 (13) |
| Ca—O1 (×2) | 3.015 (16) | (In/Sb/Sn)—O2^iv^ (×2) | 2.054 (5) |
| Ca—O1 (×2) | 3.240 (16) | Average | 2.050 |
| Ca—O2 | 2.12 (2) |  |  |
| Ca—O2^iii^ | 2.65 (3) |  |  |
| Ca—O2 | 3.18 (4) |  |  |
| Ca—O2 | 3.43 (3) |  |  |
| Average | 2.844 |  |  |
| *y* = 2, *z* = 0.5 | | | |
| Ca—O1 (×2) | 2.745 (13) | (In/Sb/Sn)—O1 (×2) | 1.989 (11) |
| Ca—O1^i^ (×2) | 2.791 (13) | (In/Sb/Sn)—O1^iii^ (×2) | 2.094 (11) |
| Ca—O1^ii^ (×2) | 2.264 (13) | (In/Sb/Sn)—O2^iii^ (×2) | 2.067 (5) |
| Ca—O1 (×2) | 3.479 (16) | Average | 2.050 |
| Ca—O2 | 2.063 (19) |  |  |
| Ca—O2^ii^ | 2.76 (2) |  |  |
| Ca—O2 | 3.09 (3) |  |  |
| Ca—O2 | 3.499 (19) |  |  |
| Average | 2.831 |  |  |
| *y* = 2, *z* = 0.75 | | | |
| Ca—O1 (×2) | 2.773 (14) | (In/Sb/Sn)—O1 (×2) | 2.006 (11) |
| Ca—O1^i^ (×2) | 2.795 (15) | (In/Sb/Sn)—O1^iv^ (×2) | 2.088 (11) |
| Ca—O1^ii^ (×2) | 2.216 (15) | (In/Sb/Sn)—O2^iv^ (×2) | 2.058 (5) |
| Ca—O1 (×2) | 3.489 (15) | Average | 2.051 |
| Ca—O2^iii^ | 2.29 (2) |  |  |
| Ca—O2^ii^ | 2.73 (2) |  |  |
| Ca—O2 | 3.02 (3) |  |  |
| Ca—O2 | 3.28 (2) |  |  |
| Average | 2.822 |  |  |
| *y* = 2, *z* = 1 | | | |
| Ca—O1 (×2) | 2.691 (17) | Sn—O1 (×2) | 2.011 (17) |
| Ca—O1^i^ (×2) | 2.877 (15) | Sn—O1^iv^ (×2) | 2.068 (17) |
| Ca—O1^ii^ (×2) | 2.217 (16) | Sn—O2^iv^ (×2) | 2.046 (5) |
| Ca—O1 (×2) | 3.466 (17) | Average | 2.042 |
| Ca—O2^iii^ | 2.32 (2) |  |  |
| Ca—O2^ii^ | 2.71 (2) |  |  |
| Ca—O2 | 3.02 (3) |  |  |
| Ca—O2 | 3.24 (2) |  |  |
| Average | 2.816 |  |  |

Symmetry codes: (i) -*x*+1/2, -*y*, *z*-1/2; (ii) *x*-1/2, -*y*+1/2, -*z*+1/2; (iii) *x*-1/2, -*y*+1/2, -*z*-1/2; (iv) -*x*+1/2, -*y*, *z*+1/2.

**Fig. S4** Cell parameters and volume variation of Ca_2_(InSb)_1-_*_z_*Sn_2_*_z_*O_6_:Fe^3+^ (*z* = 0–1).

**Fig. S5** Element mapping images of composition elements of **a** SISO:Fe^3+^, **b** CISO:Fe^3+^, and **c** CSO:Fe^3+^.

**Table S4** EXAFS fitting parameters at the Fe K-edge (*Ѕ*_0_^2^=0.75).

| Sample | Path | C.N. | *R* (Å) | *σ*^2^×10^3^ (Å^2^) | ΔE (eV) | *R* factor |
| --- | --- | --- | --- | --- | --- | --- |
| Fe foil | Fe-Fe | 8* | 2.47±0.01 | 5.0±0.9 | 7.0±1.5 | 0.002 |
|  | Fe-Fe | 6* | 2.85±0.01 | 6.5±1.9 | 5.9±2.9 |  |
| Fe | Fe-O | 7.1±0.9 | 2.04±0.01 | 6.0±1.3 | -3.3±1.4 | 0.012 |
|  | Fe-Ca | 4.2±1.8 | 3.27±0.03 | 9.9±4.4 | -1.0±3.2 |  |
|  | Fe-In/Sb | 4.5±2.1 | 3.98±0.02 | 5.2±2.9 | -1.4±2.2 |  |

*C.N.*: coordination numbers; *R*: bond distance; *σ*^2^: Debye-Waller factors; Δ*E*: the inner potential correction. *R* factor: goodness of fit. * fitting with fixed parameter.

**Fig. S6** Calculated optical bandgap values of SISO, CISO, and CSO hosts based on the diffuse reflectance spectra.

The optical band gap (*E*_g_) values of the hosts can be evaluated by the Kubelka-Munk equations:^3-4^

$$F\left( R \right)=\left( 1-R \right)^{2}/2R$$

(s1)

$$\left[ F\left( R \right)\times hv \right]^{2}=A\left( hv-E_{g} \right)$$

(s2)

where *F*(*R*) represents the absorption, *R* is the reflectance (%), and *hv* is the photon energy. Based on the DR spectra, the *E*_g_ values of SISO, CISO, and CSO hosts were determined to be 4.2, 4.5, and 4.6 eV, respectively. The large band gap indicates enough energy space for accommodating Fe^3+^ doping in the hosts.

**Fig. S7** **a** PLE spectra and **b** corresponding enlarged PLE spectra of SISO:Fe^3+^ and CISO:Fe^3+^ under 7 K.

It was found that the hosts of this series of phosphors exhibited NIR luminescence with certain intensity. As shown in Fig. S8a, SISO, CISO, and CSO hosts show broad emission peaking at 885, 935 and 1005 nm, respectively. The corresponding PLE spectra are given in Fig. S8b, all of which show a single broad excitation band in the UV region. Fig. S8c records their PL decay curves, which can be well fitted by the bi-exponential formula (*n* = 2):^5-6^

$$I=\sum_{i=1}^{n} A_{i}\exp\left( -\frac{t}{\tau_{i}} \right),\left( n=1, 2, 3, \cdots\right)$$

(s3)

where *I* is the emission intensity at time *t*, *A*_i_ is fitting constants, and *τ*_i_ is the lifetime for different components. The average lifetimes

$$\tau^{*}=\frac{A_{1}{\tau_{1}}^{2}+A_{2}{\tau_{2}}^{2}+A_{3}{\tau_{3}}^{2}+\cdots}{A_{1}\tau_{1}+A_{2}\tau_{2}+A_{3}\tau_{3}+\cdots}$$

(s4)

the lifetimes of SISO, CISO and CSO hosts were estimated to be 7.93, 8.14 and 2.85 ms, respectively. Such long lifetimes in the millisecond scale exhibit a typical feature of forbidden transition, which are in line with the lifetimes of previously reported Fe^3+^ luminescence.^7^ Moreover, some examples of Fe impurity induced host luminescence have been reported.^8-9^ Therefore, the possible contribution of unintentional Fe^3+^ dopants to the observed host luminescence should be paid attention.

Fig. S9 shows the PL spectra of SISO:*x*Fe^3+^ (*x* = 0–0.03), CISO:*x*Fe^3+^ (*x* = 0–0.03) and CSO:*x*Fe^3+^ (*x* = 0–0.02). It can be seen that each host shows identical peak profile and posision with the corresponding Fe^3+^-doped phosphors. In addition, doping Fe^3+^ can significantly increase the PL intensity of the hosts. As shown in the PLE spectra of CISO:*x*Fe^3+^ (Fig. S10), the characteristic ^6^A_1_ → ^4^T_2_ (^4^D) transition peak (~ 460 nm) of Fe^3+^ gradually appears as Fe^3+^ content increases, which is weak due to the low transition probability of forbidden 3d transitions. The strong broad band should be assigned to the O^2-^–Fe^3+^ charge transfer (CT) transition. These results support an assignment of the host luminescence to the ^4^T_1_ (^4^G) → ^6^A_1_ (^6^S) transition of Fe^3+^ impurity in the hosts. Subsequently, CISO host was taken for an example to illustrate the existence of Fe impurity, which was speculated to be from the raw materials. Hence, three different CISO hosts (host 1, host 2, and host 3) were synthesized by using raw materials of different purity (Table S4). Their PL spectra are displayed in Fig. S11. The PL intensity decreased with improving the purity of raw materials. However, even at a very high purity level of raw materials for host 3, the host luminescence still existed. ICP results (Table S5) indicate that there are traces of Fe (5 ppm) in host 3. Thus, it is reasonable to attribute the NIR luminescence of the hosts to the Fe^3+^ impurity. Table S5 also lists the Fe contents of CISO:*x*Fe^3+^ (*x* = 0, 0.001, 0.005, and 0.01), indicating that Fe is successfully incorporated in the as-syntheszed phosphors. The actually increased Fe content is consistent with the enhanced NIR emission (*x* = 0- 0.01) in Fig. S9b. From the above results, it can be inferred that the NIR luminescence of the hosts originates from the Fe^3+^ impurity.

**Fig. S8 a** PL spectra, **b** PLE spectra, and **c** PL decay curves of SISO, CISO, and CSO hosts.

**Fig. S9** PL spectra of **a** SISO:*x*Fe^3+^, **b** CISO:*x*Fe^3+^, and **c** CSO:*x*Fe^3+^.

**Fig. S10** PLE spectra of CISO:*x*Fe^3+^. The inset shows their corresponding enlarged PLE spectra in the range 380–600 nm.

**Fig. S11** PL spectra of three different CISO hosts.

**Table S5** Purity of the raw materials used to synthesize different CISO hosts.

| Sample | Raw materials | | |
| --- | --- | --- | --- |
|  | CaCO_3_ | In_2_O_3_ | Sb_2_O_3_ |
| Host 1 | 99% | 99.99% | 99.99% |
| Host 2 | 99.99% | 99.99% | 99.99% |
| Host 3 | 99.999% | 99.9999% | 99.999% |

**Table S6** Detected content (%) of Fe in CISO:*x*Fe^3+^ by ICP-AES.

| Sample | Fe (%) |
| --- | --- |
| CISO (host 3) | 0.0005 |
| CISO (host 1) | 0.054 |
| CISO:0.001Fe^3+^ | 0.062 |
| CISO:0.005Fe^3+^ | 0.069 |
| CISO:0.01Fe^3+^ | 0.166 |

**Fig. S12** Luminescence decay curves of Sr_2-_*_y_*Ca*_y_*InSbO_6_:Fe^3+^ (*y* = 0, 1, and 2).

**Table S7** Lists of the fitting parameters of the decay curves for Sr_2-_*_y_*Ca*_y_*InSbO_6_:Fe^3+^ (*y* = 0, 1, and 2).

| *y* | A_1_ | 𝜏_1_ | A_2_ | 𝜏_2_ | 𝜏_av_ | *chi*^2^ | *R*^2^ |
| --- | --- | --- | --- | --- | --- | --- | --- |
| 0 | 0.4152 | 5.09 | 0.5659 | 9.58 | 8.32 | 2.08×10^-4^ | 0.9933 |
| 1 | 0.1468 | 2.27 | 0.8851 | 7.59 | 7.34 | 2.89×10^-4^ | 0.9906 |
| 2 | 0.1839 | 0.88 | 0.8819 | 8.15 | 7.99 | 2.20×10^-4^ | 0.9926 |

**Fig. S13 a** PL spectrum of CISO:Fe^3+^ measured at 7 K. **b** Normalized PLE spectra of CISO:Fe^3+^ monitored at different emission wavelengths, measured at 7 K. **c** Luminescence decay curves of CISO:Fe^3+^ monitored at different emission wavelengths at 7 K. **d** TRPL spectra of CISO:Fe^3+^ measured at 7 K.

**Fig. S14** PL intensity of Ca_2_(InSb)_1-_*_z_*Sn_2_*_z_*O_6_:Fe^3+^ (*z* = 0–1).

**Fig. S15** TRPL spectra of CSO:Fe^3+^.

**Fig. S16** Dependence of log(*I*/*x*) on log(*x*) in CSO:Fe^3+^.

**Table S8** Lists of the fitting parameters of the decay curves for Ca_2_(InSb)_1-z_Sn_2z_O_6_:Fe^3+^ (*z* = 0 and 1).

| *z* | A_1_ | | 𝜏_1_ | | A_2_ | | 𝜏_2_ | | 𝜏_av_ | | *chi*^2^ | | *R*^2^ | |
| --- | --- | --- | --- | --- | --- | --- | --- | --- | --- | --- | --- | --- | --- | --- |
| Mono-exponential fitting | | | | | | | | | | | | | | |
| 0 | | 0.9243 | | 7.79 | | / | | / | | / | | 2.42×10^-4^ | | 0.9919 |
| 1 | | 0.9204 | | 2.66 | | / | | / | | / | | 2.35×10^-4^ | | 0.9862 |
| Bi-exponential fitting | | | | | | | | | | | | | | |
| 0 | | 0.1839 | | 0.88 | | 0.8819 | | 8.15 | | 7.99 | | 2.20×10^-4^ | | 0.9926 |
| 1 | | 1.2499 | | 0.38 | | 0.7314 | | 3.24 | | 2.76 | | 0.67×10^-4^ | | 0.9961 |

**Temperature-dependent luminescent properties:** The temperature-dependent PL spectra of the as-prepared SISO:Fe^3+^, CISO:Fe^3+^, and CSO:Fe^3+^ phosphors are displayed in Fig. S16a-c. At 398 K, the integrated PL intensity decreased to 44%, 36%, and 24% of the initial intensity at 298 K for SISO:Fe^3+^, CISO:Fe^3+^, and CSO:Fe^3+^, respectively (Fig. S16d). Although CISO:Fe^3+^ show slower thermal quenching than SISO:Fe^3+^ and CSO:Fe^3+^, its thermal stability should be further improved for practical applications. The PL intensity can be fitted by the Arrhenius equation (Fig. S17):^10-12^

$$\ln\left( \frac{I_{0}}{I_{T}}-1 \right)=\ln A-\frac{E_{a}}{kT}$$

(s5)

where *I*_0_ and *I_T_* represent the PL intensity at 298 K and temperature *T*, respectively. *A* is a constant, *k* is the Boltzmann’s constant, and *E*_a_ is the activation energy for thermally activated crossover process, which results in the thermal quenching. Accordingly, the *E*_a_ values of SISO:Fe^3+^, CISO:Fe^3+^, and CSO:Fe^3+^ were evaluated to be 0.29 eV, 0.31 eV, and 0.36 eV, respectively. The larger activation energy of CISO:Fe^3+^ than SISO:Fe^3+^ can be responsible for the better thermal stability of CISO:Fe^3+^ than SISO:Fe^3+^. Although CSO:Fe^3+^  has a higher activation energy, it exhibits serious thermal quenching, which may because of its large stokes shift. The stokes shift is closely related to structural rigidity.^13^ Phosphors with large stokes shift usually have weak structural rigidity, thereby leading to the poor thermal stability.^11^ Fig. S18 shows the temperature-dependent decay curves of CISO:Fe^3+^ from 7 to 423 K. With the increase of temperature, the lifetime was shortened from 15.6 to 2.1 ms, ascribing to the lattice thermal vibration induced nonradiative transition.

**Fig. S17** Temperature-dependent PL spectra of **a** SISO:Fe^3+^, **b** CISO:Fe^3+^, and **c** CSO:Fe^3+^. **d** Emission intensity of SISO:Fe^3+^, CISO:Fe^3+^, and CSO:Fe^3+^ as a function of temperature.

**Fig. S18** Dependence of ln(*I*_0_/*I_T_*-1) on (1/*kT*) according to the temperature-dependent PL spectra of **a** SISO:Fe^3+^, **b** CISO:Fe^3+^, and **c** CSO:Fe^3+^.

**Fig. S19** Temperature-dependent decay curves of CISO:Fe^3+^.

**Fig. S20** IQE measurements for **a** SISO:Fe^3+^, **b** CISO:Fe^3+^, **c** La_3_Ga_5_GeO_14_:Cr^3+^, and **d** NaScGe_2_O_6_:Cr^3+^. The insets show the amplified emission spectra.

We have measured the internal quantum efficiency (*η*_i_) using the integrated sphere on FLS1000 instrument, and white BaSO_4_ powder was used as a reference. The internal quantum efficiencies of SISO:Fe^3+^ and CISO:Fe^3+^ are 48% and 87%, respectively. The absorption efficiency (*α*_abs_) and external quantum efficiency (*η*_e_) can be calculated by using the following equations:^14^

$$\alpha_{\mathrm{abs}}=\frac{\alpha}{\delta}=\frac{\int E_{R}-\int E_{S}}{\int E_{R}}$$

$$\eta_{e}=\eta_{i}*\alpha_{\mathrm{abs}}$$

where *δ* is the number of total photons excited by the light source and *α* is the number of photons absorbed by the sample. *E_R_* is the spectrum of the excitation light with BaSO_4_ in the sphere; *E*_S_ is the spectrum of the excitation light with the sample in the sphere; all the spectra were collected using the sphere. Accordingly, the absorption efficiency of SISO:Fe^3+^ and CISO:Fe^3+^ are 73% and 78%, respectively, and the external quantum efficiency of SISO:Fe^3+^ and CISO:Fe^3+^ are 35% and 68%, respectively.

**Fig. S21** XRD patterns of NaScGe_2_O_6_:Cr^3+^ and La_3_Ga_5_GeO_14_:Cr^3+^.

**Fig. S22** PL spectra of La_3_Ga_5_GeO_14_:Cr^3+^, NaScGe_2_O_6_:Cr^3+^, SISO:Fe^3+^, and CISO:Fe^3+^.

**Fig. S23** Emission spectra of the as-fabricated pc-LEDs using **a** SISO:Fe^3+^, **b** CISO:Fe^3+^, and **c** CSO:Fe^3+^ phosphors.

**Fig. S24** NIR output power under different driving currents of the as-fabricated pc-LEDs using SISO:Fe^3+^, CISO:Fe^3+^, and CSO:Fe^3+^ phosphors.

**Fig. S25** Emission spectra of SISO:Fe^3+^, CISO:Fe^3+^, and CSO:Fe^3+^ phosphors, and commercial 940 nm chip.

**References:**

(1) Shaheen, R. & Bashir, J. Crystal structure of A_2_InSbO_6_ (A=Ca, Sr, Ba) ordered double perovskites. *Solid State Sci.* **12**, 605-609 (2010).

(2) Zhao, J. Ross, N. L. & Angel, R. J. Tilting and distortion of CaSnO_3_ perovskite to 7 GPa determined from single-crystal X-ray diffraction. *Phys. Chem. Miner.* **31**, 299-305 (2004).

(3) Wei, Y. et al. Highly Efficient Blue Emission and Superior Thermal Stability of BaAl_12_O_19_:Eu^2+^ Phosphors Based on Highly Symmetric Crystal Structure. *Chem. Mater.* **30**, 2389-2399 (2018).

(4) Liu, D. et al. Yellow/Orange-Emitting ABZn_2_Ga_2_O_7_:Bi^3+^ (A = Ca, Sr; B = Ba, Sr) Phosphors: Optical Temperature Sensing and White Light-Emitting Diode Applications. *Chem. Mater.* **32**, 3065-3077 (2020).

(5) Qiao, J., Zhou, G., Zhou, Y., Zhang, Q. & Xia, Z. Divalent europium-doped near-infrared-emitting phosphor for light-emitting diodes. *Nat. Commun.* **10**, 5267 (2019).

(6) Liang, S. et al. New Insight for Luminescence Tuning Based on Interstitial sites Occupation of Eu^2+^ in Sr_3_Al_2−_*_x_*Si*_x_*O_5−_*_x_*N*_x_*Cl_2_ (*x*= 0-0.4). *Adv. Opt. Mater.* **6***,* 1800940 (2018)*.*

(7) Lin, L. Comment on “Oxygen-Vacancy-Induced Midgap States Responsible for the Fluorescence and the Long-Lasting Phosphorescence of the Inverse Spinel Mg(Mg,Sn)O_4_”. *Chem. Mater.* **32**, 7564-7567 (2020).

(8) Sosman, L. P., López, A., Pedro, S. S. & Papa, A. R. R. Photoluminescence of the Mg_2_Al_4_Si_5_O_18_-Al_2_O_3_-MgAl_2_O_4_-SiO_2_ ceramic system containing Fe^3+^ and Cr^3+^ as impurity ions. *Opt. Mater.* **76**, 353-358 (2018).

(9) Baur, J. et al. Photoluminescence of residual transition metal impurities in GaN. *Appl. Phys. Lett.* **67**, 1140-1142 (1995).

(10) Mao, M. et al. Broadband near-infrared (NIR) emission realized by the crystal-field engineering of Y_3-_*_x_*Ca*_x_*Al_5-_*_x_*Si*_x_*O_12_:Cr^3+^ (*x*=0-2.0) garnet phosphors. *J. Mater. Chem. C* **8**, 1981-1988 (2020).

(11) Zhao, M. et al. Discovery of New Narrow‐Band Phosphors with the UCr4C4‐Related Type Structure by Alkali Cation Effect. *Adv. Opt. Mater.* **7***,* 1801631 (2019).

(12) Qiao, J. et al. Eu^2+^ Site Preferences in the Mixed Cation K_2_BaCa(PO_4_)_2_ and Thermally Stable Luminescence. *J. Am. Chem. Soc.* **140**, 9730-9736 (2018).

(13) Denault, K. A. et al. Average and Local Structure, Debye Temperature, and Structural Rigidity in Some Oxide Compounds Related to Phosphor Hosts. *ACS Appl. Mater. Interfaces* **7**, 7264-7272 (2015).

(14) Yang, Z. et al. Giant Red‐Shifted Emission in (Sr,Ba)Y_2_O_4_:Eu^2+^ Phosphor Toward Broadband Near‐Infrared Luminescence. *Adv. Funct. Mater.* **32**, 2103927 (2021).
