# Supplementary material for: Highly efficient Fe3+-doped A2BB′O6 (A = Sr2+, Ca2+; B, B′ = In3+, Sb5+, Sn4+) broadband near-infrared-emitting phosphors for spectroscopic analysis
Source: Light Sci Appl. 2022 Apr 27;11:112. doi: 10.1038/s41377-022-00803-x (PMC9046267; doi:10.1038/s41377-022-00803-x)
Supplement: Supplementary file 2 — 文章保密与发表审查单 [file 41377_2022_803_MOESM2_ESM.pdf]

文章保密与发表审查单

承 诺 书

此文章不涉密且不存在造假、抄袭、一稿多投等学术不端行为，特此承诺。

第一（通讯）作者签字： 刘冬杰

2021年 11 月 24日

《Light: Science & Applications》编辑部：

刘冬杰，李国岗*，党佩佩，张倩倩，魏忆，邱磊，Maxim S. Molokeev，连洪洲，尚蒙蒙，林君* 作者（需按正式发表文章署名顺序，填写全部作者姓名）为你刊撰写的文章（题目：Highly efficient Fe^{3+}-doped $A_2BB'O_6$ (A = Sr^{2+}, Ca^{2+}; B, B' = In^{3+}, Sb^{5+}, Sn^{4+}) broadband near-infrared-emitting phosphors for spectroscopic analysis），经审查，未发现该文章存在涉密内容和造假、抄袭、一稿多投等学术不端现象。该文章一经录用，其数字化复制权、发行权、汇编权及信息网络传播权将转让予《Light: Science & Applications》编辑部。

导师（课题负责人）签字　　　　通讯或第一作者的单位或单位保密机构盖章

2021 年 11月24日　　　　2021 年 11 月24日
